# Sleep structure and sleepiness in chronic fatigue syndrome with or without coexisting fibromyalgia

**DOI:** 10.1186/ar2425

**Published:** 2008-05-13

**Authors:** Fumiharu Togo, Benjamin H Natelson, Neil S Cherniack, Jennifer FitzGibbons, Carmen Garcon, David M Rapoport

**Affiliations:** 1 Pain and Fatigue Study Center, Department of Neurosciences, University of Medicine and Dentistry of New Jersey (UMDNJ)-New Jersey Medical School, 30 Bergen Street, Newark, NJ 07103, USA; 2 Department of Work Stress Control, Japan National Institute of Occupational Safety and Health, 6-21-1 Nagao, Tama-ku, Kawasaki, 214-8585, Japan; 3 Pain and Fatigue Study Center, Department of Medicine, UMDNJ-New Jersey Medical School, 30 Bergen Street, Newark, NJ 07103, USA; 4 Department of Medicine, Division of Pulmonary and Critical Care Medicine, New York University School of Medicine, 462 First Avenue, New York, NY 10016, USA

## Abstract

**Introduction:**

We evaluated polysomnograms of chronic fatigue syndrome (CFS) patients with and without fibromyalgia to determine whether patients in either group had elevated rates of sleep-disturbed breathing (obstructive sleep apnea or upper airway resistance syndrome) or periodic leg movement disorder. We also determined whether feelings of unrefreshing sleep were associated with differences in sleep architecture from normal.

**Methods:**

We compared sleep structures and subjective scores on visual analog scales for sleepiness and fatigue in CFS patients with or without coexisting fibromyalgia (n = 12 and 14, respectively) with 26 healthy subjects. None had current major depressive disorder, and all were studied at the same menstrual phase.

**Results:**

CFS patients had significant differences in polysomnograpic findings from healthy controls and felt sleepier and more fatigued than controls after a night's sleep. CFS patients as a group had less total sleep time, lower sleep efficiency, and less rapid eye movement sleep than controls. A possible explanation for the unrefreshing quality of sleep in CFS patients was revealed by stratification of patients into those who reported more or less sleepiness after a night's sleep (a.m. sleepier or a.m. less sleepy, respectively). Those in the sleepier group reported that sleep did not improve their symptoms and had poorer sleep efficiencies and shorter runs of sleep than both controls and patients in the less sleepy group; patients in the less sleepy group reported reduced fatigue and pain after sleep and had relatively normal sleep structures. This difference in sleep effects was due primarily to a decrease in the length of periods of uninterrupted sleep in the a.m. sleepier group.

**Conclusion:**

CFS patients had significant differences in polysomnographic findings from healthy controls and felt sleepier and more fatigued than controls after a night's sleep. This difference was due neither to diagnosable sleep disorders nor to coexisting fibromyalgia but primarily to a decrease in the length of periods of uninterrupted sleep in the patients with more sleepiness in the morning than on the night before. This sleep disruption may explain the overwhelming fatigue, report of unrefreshing sleep, and pain in this subgroup of patients.

## Introduction

Chronic fatigue syndrome (CFS) is a medically unexplained condition occurring mostly in women and is characterized by persistent or relapsing fatigue that lasts at least 6 months and substantially interferes with normal activities. In addition to severe fatigue, one of the symptoms used for diagnosing CFS is unrefreshing sleep, and, in fact, this sleep-related problem is the most common complaint among patients with severe medically unexplained fatigue [1]. An obvious possibility is that patients with this problem have an underlying sleep disorder or substantial amounts of interrupted sleep which may be responsible for the genesis of the illness. This idea was supported by a recent longitudinal study that indicated that 20% of a carefully delineated group of CFS patients were found to have sleep apnea or narcolepsy, exclusions for the diagnosis of CFS [2].

A number of reports of polysomnography in CFS patients were remarkable for finding high rates of these sleep disorders plus periodic leg movement (PLM) disorder [3-5], whereas several recent studies have found rates to be the same as in controls [6,7]. One possible reason for this discrepancy is the existence of coexisting fibromyalgia (FM). None of these previous papers stratified their patient sample as to the existence of coexisting FM, and recent data indicate substantial amounts of sleep-disturbed breathing in patients with this disorder [8,9]. FM is a medically unexplained syndrome characterized by four quadrant pain and multiple tender points and frequently occurs in conjunction with CFS [10]. Hence, we evaluated polysomnograms (PSGs) of CFS patients with and without FM to determine whether patients in either group had elevated rates of sleep-disturbed breathing (obstructive sleep apnea or upper airway resistance syndrome) or PLM disorder.

Another important issue was whether CFS patients would show abnormalities in their sleep architecture even if clinical sleep disorders were not present. Two studies have been done on patients with 'pure' CFS (that is, in patients with neither sleep disorder nor evidence of current depression, another illness that can interfere with sleep) with differing results: one reported low sleep efficiency with increased periods of wakefulness in CFS [11] and the second found normal sleep architecture [12]. We decided to extend these studies and to determine whether the patient's subjective response to sleep, another source of variability in a clinical sample, might correlate with or predict sleep disturbance. Therefore, we also determined whether feelings of unrefreshing sleep were associated with differences in sleep architecture from normal.

## Materials and methods

### Subjects

The subjects were 62 women (32 with CFS and 30 healthy controls) ranging in age from 27 to 56 years. Subjects older or younger than those selected were excluded because of age effects on sleep. There were no differences in age or body mass index between patients or controls (Table 1). Subjects were recruited either from our data set of prior research subjects or from the clinical practice of author BHN, who specializes in the care of these patients. Other patients were referred by their physician or were self-referred based on media reports about our research. All subjects initially completed an extensive health screening form (available at [13]) that over the years has proven effective in identifying CFS patients (approximately 5% margin of error). This screening vehicle was also used to exclude patients taking antidepressants, opiates, steroids, hypnotics, and other sedatives, including benzodiazepines. Patients screening positive for CFS and controls indicating their health to be excellent or good – not fair or poor – arrived at our center, where they gave their informed consent and were approved by the medical school's institutional review board to participate in this research (n = 53 patients and 42 healthy controls).

**Table 1:** Selected sleep stage variables in healthy controls and chronic fatigue syndrome patients without sleep abnormalities

	Healthy	Chronic fatigue syndrome
Number	26	26
Age, years	38 ± 8	39 ± 8
Body mass index, kg/m^2^	24.4 ± 4.8	24.9 ± 5.2
CES-D score	8 ± 7	17 ± 8^a^
Sleep structure		
Time in bed, minutes	453 ± 33	438 ± 42
Total sleep time, minutes	385 ± 39	351 ± 54^a^
Sleep efficiency^b^, percentage	85 ± 8	80 ± 10^a^
Number of arousals per hour	8.2 ± 5.2	5.7 ± 4.9
Wakefulness, minutes	51 ± 38	61 ± 32
Stage 1, minutes	44 ± 18	33 ± 17^a^
Wakefulness plus stage 1, minutes	96 ± 43	94 ± 35
Stage 2, minutes	225 ± 36	198 ± 33^a^
Stage 3, minutes	29 ± 20	46 ± 23^a^
Stage 4, minutes	5 ± 11	10 ± 15
Slow-wave sleep (stage 3 + 4), minutes	34 ± 26	56 ± 34
Stage REM, minutes	84 ± 25	64 ± 26^a^
Sleep latency^c^, minutes	16 ± 18	26 ± 26
REM latency^d^, minutes	105 ± 49	127 ± 65
Median duration of sleep runs, minutes	15.6 ± 18.4	8.9 ± 5.7^a^
Likert scale (0–15.5)		
Sleepiness		
Evening	6 ± 4	9 ± 5^a^
Morning	1 ± 2^e^	8 ± 4^a^
Fatigue		
Evening	4 ± 4	11 ± 3^a^
Morning	2 ± 2^e^	9 ± 4^a^
Pain		
Evening	1 ± 2	7 ± 5^a^
Morning	0 ± 1	7 ± 5^a^
Feeling blue		
Evening	1 ± 2	2 ± 2
Morning	0 ± 0^e^	1 ± 3^a^

Values are presented as mean ± standard deviation. ^a^Significantly different from healthy controls (*P *< 0.05, non-paired *t *test). ^b^Total sleep time/time in bed × 100%. ^c^Time from lights out to sleep onset. ^d^Time from lights out to first epoch of stage REM. ^e^Significantly different from evening (*P *< 0.05, paired *t *test). CES-D, Centers for Epidemiological Study-Depression; REM, rapid eye movement.

Subsequently, each research subject underwent a complete medical history and physical examination, including a tender point evaluation, and a psychiatric diagnostic interview (Quick Diagnostic Interview Schedule, Q-DIS), all of which were administered by the study's advanced practice nurse (JF) under the supervision of BHN. The psychiatric interview [14] was used to identify DSM-IV (*Diagnostic and Statistical Manual of Mental Disorders, Fourth Edition*)-based exclusionary disorders, including schizophrenia, eating disorders, substance abuse, or bipolar disorder [15], as well as major depressive disorder, a psychiatric disorder that can disrupt sleep [16]. Finally, a set of blood tests was done to identify medical causes of fatigue. These tests included complete blood count with sedimentation rate, liver and thyroid function tests, Lyme antibody, anti-nuclear antibodies, rheumatoid factor, and C-reactive protein.

Following this evaluation, 21 patients and 12 healthy subjects were dropped from further study for the following reasons: inadequate criteria for CFS, 3 patients; use of exclusionary drugs, 6 patients; previously unappreciated medical illness, 1 patient and 2 controls; current depression, 5 patients; obesity, 1 patient; abnormal labs, 1 patient and 5 controls; moved or no longer interested, 3 patients and 2 controls; and technical or other problem, 1 patient and 3 controls. The remaining patients all fulfilled the 1994 case definition for CFS [15]; of these patients, 14 also fulfilled the American College of Rheumatology criteria (1990) for FM [17].

### Procedures

Subjects were instructed to refrain from alcohol and caffeine ingestion and to avoid engaging in prolonged and/or strenuous exercise in the daytime of study nights; thereafter, subjects underwent one night of PSG recording in a quiet, shaded hospital room. Subjects went to bed at their usual bedtime (patients: 11:40 p.m. ± 1 hour 9 minutes; controls: 11:15 p.m. ± 1 hour 26 minutes) and slept until 7:15 to 8 a.m. the next morning.

### Measurements

Subjects underwent full nocturnal polysomnography (Compumedics, Charlotte, NC, USA) consisting of electroencephalogram (EEG) (C3/A2, O1/A2, and FZ/A2), electrooculogram (EOG), submental electromyogram (EMG), anterior tibialis EMG, a lead II electrocardiogram (ECG), thoracic and abdominal motion, airflow using a nasal cannula/pressure transducer and an oral thermistor, and pulse oximetry. Analog signals for EEG, EOG, EMG, ECG, thoracic and abdominal motion, airflow, and pulse oximetry were processed on a real-time basis, using a Dell personal computer (Dell, Round Rock, TX, USA). Sleep was scored every 30 seconds by a single scorer according to standard criteria of Rechtschaffen and Kales [18]. Sleep onset was defined as the first three consecutive epochs of sleep stage 1 or the first epoch of other stages of sleep. An arousal was defined according to standard criteria of the American Academy of Sleep Medicine [19] as a return to alpha- or fast-frequency EEG activity, well differentiated from the background, lasting at least 3 seconds but no more than 15 seconds. Respiratory events were defined as any combination of apnea and hypopnea lasting at least 10 seconds or airflow suggesting flow limitation lasting at least 10 seconds associated with an arousal. Apnea was defined as a reduction in airflow to less than 10% of waking level in the nasal cannula and absent airflow in the oral thermistor, and hypopnea was defined as a decrease in inspiratory airflow to less than 50% of waking levels. Flow limitation was considered to occur when there were two or more consecutive breaths (for an event duration generally greater than or equal to 10 seconds) that had a flattened or non-sinusoidal appearance but had peak inspiratory amplitudes that did not meet the greater than 50% reduction requirement of hypopnea. These events were required to end abruptly with a return to breaths with sinusoidal shape. The respiratory disturbance index (RDI) was defined as the total number of apneas, hyponeas, and flow limitation events per hour of sleep [20]. The RDI including the flow limitation events terminated by arousal has been previously shown to be nearly identical to the number of esophageal manometry events terminated by arousal, which have been called respiratory effort-related arousals [20]. Based on results by Ayappa and colleagues [20], it was assumed that an RDI of greater than or equal to 18 events per hour was sufficient to account for excessive daytime sleepiness on the basis of sleep-disordered breathing, and the diagnosis of sleep-disturbed breathing was then made for patients and healthy controls with this finding. PLMs were defined as four or more consecutive involuntary leg movements per hour during sleep, lasting 0.5 to 5.0 seconds, with an intermovement interval of 5 to 90 seconds. Patients were labeled as having Periodic Leg Movements in Sleep (PLMS) syndrome when the number of PLMs per hour (index) was greater than 5.

### Sleep continuity

Sleep continuity was evaluated by generating a nonparametric survival curve calculated from the combined data within each group [21,22] of the varying durations of sequential sleep runs (that is, continuous epochs of sleep separated from one another by epochs of wakefulness) and was expressed as the median duration of all continuous epochs scored as sleep in each subject. A run of sleep was defined using the sequence of epoch-based sleep stages represented in the hypnogram. A run began with a change from waking to any stage of sleep. A sleep run continued until there was a change from any stage of sleep to waking. To compare sleep continuity between groups, all data from all subjects in each group were pooled and a group survival curve was generated using standard statistical techniques that take into account the multiple runs of sleep in each subject [21,22]; this method was derived from an earlier one [23].

### Subjective test

Subjects were asked to indicate their levels of perceived sleepiness, fatigue, pain, and feeling blue on separate 15.5 cm visual analog scales (0 to 15.5) given to them immediately before lights out and after awakening.

### Depressed mood

The Centers for Epidemiological Study-Depression (CES-D) scale was used as an indicator of depressed mood. This 20-item scale required respondents to rate how often certain symptoms occurred during the past week on a scale from rarely or none (0) to most all the time (3). Items were summed to yield a total score. The higher the value, the more depressed the mood.

### Statistical analyses

We dichotomized patients' data based on their self-reported sleepiness before and after sleep. We labeled those with more sleepiness in the morning than on the night before as 'a.m. sleepier' and those with less sleepiness in the morning than on the night before as 'a.m. less sleepy'. Changes of sleepiness before and after sleep as well as changes in the other variables captured via visual analog scale were assessed using the paired *t *test (Tables 1, 2, 3). Differences in measured variables between groups were assessed using the non-paired *t *test (Tables 1 and 2) or analysis of variance (Table 3). *Post hoc *analyses used Tukey Student range tests to adjust for multiple comparisons (Table 3). Interrelationships between subjective scales/psychological data and sleep structure were tested by simple Pearson correlation coefficients, and interrelationships between subjective scales were tested by least squares regression analyses. A *P *value of less than 0.05 was considered statistically significant.

**Table 2:** Selected sleep stage variables in chronic fatigue syndrome patients with and without coexisting fibromyalgia

	CFS alone	CFS + FM
Number	14	12
Age, years	37 ± 9	41 ± 6
Body mass index, kg/m^2^	23.3 ± 5.0	26.7 ± 6.0
CES-D score	17 ± 7	18 ± 10
Sleep structure		
Time in bed, minutes	440 ± 40	436 ± 46
Total sleep time, minutes	346 ± 60	358 ± 49
Sleep efficiency, percentage	78 ± 10	82 ± 9
Number of arousals per hour	6.2 ± 6.2	5.2 ± 3.2
Wakefulness, minutes	63 ± 27	59 ± 39
Stage 1, minutes	38 ± 20	27 ± 10
Wakefulness plus stage 1, minutes	101 ± 28	86 ± 41
Stage 2, minutes	203 ± 35	192 ± 30
Stage 3, minutes	35 ± 23	59 ± 16
Stage 4, minutes	10 ± 16	11 ± 15
Slow-wave sleep (stage 3 + 4), minutes	45 ± 34	70 ± 28
Stage REM, minutes	60 ± 22	69 ± 30
Sleep latency, minutes	31 ± 27	20 ± 24
REM latency, minutes	149 ± 77	101 ± 37
Median duration of sleep runs, minutes	8.5 ± 5.9	9.5 ± 5.5
Likert scale (0–15.5)		
Sleepiness		
Evening	10 ± 5	8 ± 3
Morning	9 ± 4	7 ± 3
Fatigue		
Evening	10 ± 3	11 ± 3
Morning	9 ± 4	10 ± 3
Pain		
Evening	6 ± 6	9 ± 3
Morning	6 ± 5	9 ± 4
Feeling blue		
Evening	1 ± 1	2 ± 3
Morning	1 ± 3	1 ± 2

Values are presented as mean ± standard deviation. CES-D, Centers for Epidemiological Study-Depression; CFS, chronic fatigue syndrome; FM, fibromyalgia; REM, rapid eye movement.

**Table 3:** Selected sleep stage variables in healthy controls and chronic fatigue syndrome patients who were either less sleepy or sleepier after polysomnography

	Healthy	CFS a.m. less sleepy^a^	CFS a.m. sleepier^a^
Number	26	14	12
Age, years	37 ± 8	39 ± 8	39 ± 8
Body mass index, kg/m^2^	24.0 ± 4.4	24.1 ± 5.7	25.8 ± 4.7
CES-D score	8 ± 7	20 ± 8^b^	14 ± 7^b,c^
Sleep structure			
Time in bed, minutes	453 ± 33	433 ± 41	445 ± 44
Total sleep time, minutes	385 ± 38	350 ± 50^b^	353 ± 61^b^
Sleep efficiency, percentage	85 ± 8	81 ± 9	79 ± 10^b^
Number of arousals per hour	8.3 ± 5.2	4.8 ± 3.7	6.7 ± 6.1
Wakefulness, minutes	52 ± 38	64 ± 33	61 ± 35
Stage 1, minutes	45 ± 19	32 ± 12	34 ± 22
Wakefulness plus stage 1, minutes	96 ± 44	96 ± 34	95 ± 37
Stage 2, minutes	222 ± 34	195 ± 29^b^	202 ± 38
Stage 3, minutes	30 ± 22	50 ± 20^b^	41 ± 26
Stage 4, minutes	5 ± 11	13 ± 15	7 ± 15
Slow-wave sleep (stage 3 + 4), minutes	35 ± 26	63 ± 30^b^	48 ± 37
Stage REM, minutes	83 ± 24	60 ± 18^b^	69 ± 33
Sleep latency, minutes	16 ± 19	20 ± 20	32 ± 31^b^
REM latency, minutes	107 ± 49	128 ± 64	126 ± 69
Median duration of sleep runs, minutes	15.6 ± 18.4	10.0 ± 6.7	7.9 ± 3.3^b^
Likert scale (0–15.5)			
Sleepiness			
Evening	6 ± 4	12 ± 2^b^	6 ± 5^c^
Morning	1 ± 2^d^	7 ± 4^b,d^	10 ± 2^b-d^
Fatigue			
Evening	4 ± 4	11 ± 3^b^	10 ± 3^b^
Morning	1 ± 2^d^	8 ± 4^b,d^	11 ± 3^b,c^
Pain			
Evening	1 ± 2	8 ± 4^b^	7 ± 5^b^
Morning	0 ± 1	6 ± 4^b,d^	9 ± 4^b,c^
Feeling blue			
Evening	1 ± 2	2 ± 3	1 ± 1
Morning	0 ± 0^d^	1 ± 3	1 ± 3

Values are presented as mean ± standard deviation. ^a^Data dichotomized based on difference between daytime and nighttime self-reported ratings of sleepiness (*P *< 0.05, analysis of variance [ANOVA]). ^b^Significantly different from healthy controls. ^c^Significantly different from CFS in the a.m. less sleepy group (*P *< 0.05, ANOVA). ^d^Significantly different from evening (*P *< 0.05, paired *t *test). CES-D, Centers for Epidemiological Study-Depression; CFS, chronic fatigue syndrome; REM, rapid eye movement.

## Results

Evaluation of the PSG led us to exclude 10 subjects with clinically significant sleep abnormalities: 3 controls with RDIs of 26, 22.4, and 18/hour, 1 CFS patient with an RDI of 22.1/hour, 1 CFS/FM patient with an RDI of more than 40/hour, and 1 control, 3 CFS patients, and 1 CFS/FM patient with PLMs. We included 1 CFS patient and 3 healthy controls with RDIs of 10.4, 10.8, 10.1, and 9.5/hour, respectively, as these fall within the range seen in asymptomatic normal subjects in the study by Ayappa and colleagues [20]. This left a total of 26 CFS patients, 12 with comorbid FM, and 26 healthy control subjects.

Table 1 depicts the key PSG measures of the healthy controls and CFS patients. Total sleep time was significantly longer for healthy controls than patients as were the total durations of stage 1, stage 2, and rapid eye movement (REM) sleep, whereas total duration of wakefulness did not differ between healthy controls and patients. As a result, patients had a significantly lower sleep efficiency (that is, the percentage of the total time asleep after falling asleep relative to the time spent in bed) than healthy controls. However, sleep latency, defined as the time from lights out to the first three consecutive epochs of sleep stage 1 or the first epoch of other stages of sleep (that is, sleep onset), and total duration of slow-wave sleep (SWS) (that is, the sum of stage 3 and 4 sleep) did not differ significantly between groups. Data were also evaluated based on whether the patient had CFS alone or CFS plus FM (Table 2). Patients with CFS plus FM had sleep structures similar to those of patients with CFS alone.

Table 1 also shows that sleepiness, fatigue, and pain before and after the PSG night were significantly higher in patients than healthy controls. Values for subjective a.m. sleepiness, fatigue, and feeling blue decreased compared with the evening in healthy controls, whereas none of these decreased for patients.

For patients, self-rated sleepiness, fatigue, and pain before sleep correlated positively with sleep efficiency (r = 0.39, 0.59, 0.57; *P *< 0.05) and duration of stage 4 sleep (r = 0.48, 0.42, 0.56; *P *< 0.05) and negatively with sleep latency (r = -0.40, -0.42, -0.40; *P *< 0.05); self-rated fatigue and pain correlated negatively with durations of wakefulness after sleep onset (r = -0.50, -0.43; *P *< 0.05) and wakefulness plus stage 1 sleep (r = -0.50, -0.57; *P *< 0.05). Self-rated fatigue correlated negatively with REM latency (r = -0.49; *P *< 0.05). Self-rated pain correlated positively with total sleep time (r = 0.49; *P *< 0.05) and durations of stage 3 sleep (r = 0.49; *P *< 0.05) and SWS (r = 0.59; *P *< 0.05). Moreover, change in self-rated sleepiness and fatigue over the night correlated positively with sleep latency (r = 0.39, 0.49; *P *< 0.05) and negatively with sleep efficiency (r = -0.41, -0.54; *P *< 0.05). Changes in self-rated fatigue over the night correlated negatively with total sleep time (r = -0.39; *P *< 0.05) and total duration of stage 3 sleep (r = -0.41; *P *< 0.05). No significant relations were found among any of these variables for the healthy control group. When we looked at correlations between self-rated variables reported after sleep and sleep stage variables, none was significant except that self-rated sleepiness after sleep correlated positively with duration of stage 2 sleep (r = 0.48; *P *< 0.05) for the healthy controls.

**Figure 1:**
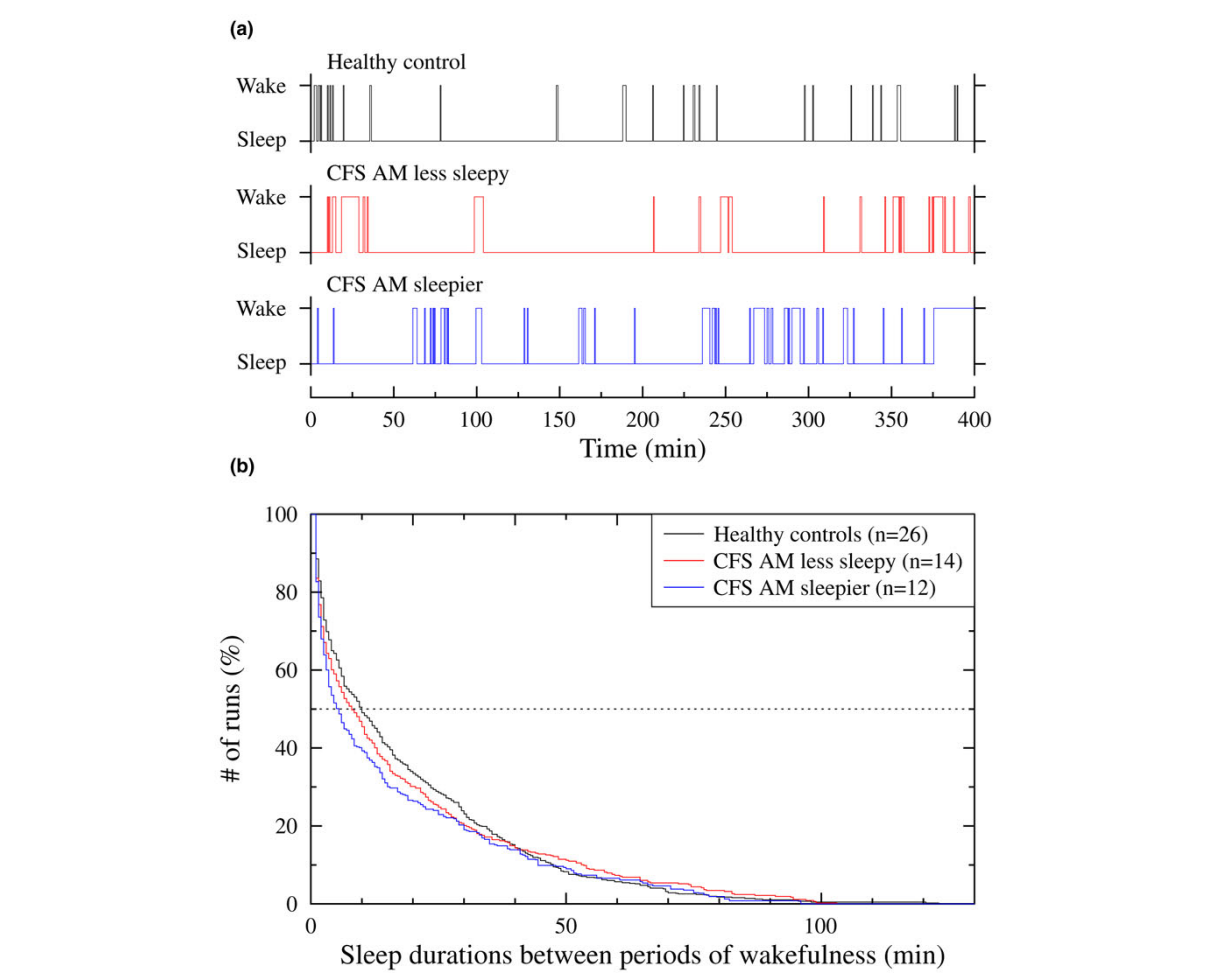
Sleep-wake patterns and survival curves for the duration of every episode of sleep. **(a) **Representative sleep-wake patterns from one healthy control, one patient in the a.m. less sleepy group, and one patient in the a.m. sleepier group. In contrast to the control and a.m. less sleepy patient, the a.m. sleepier patient shows clustering of her arousals, which is documented in the accompanying panel. **(b) **Survival curves of every episode of sleep (that is, a bout of sleep preceded and followed by periods of wakefulness) for controls and patients in the a.m. less sleepy and a.m. sleepier groups for whole-night hypnograms stratified by the duration of the sleep episode. To compare sleep continuity between groups, all data from all subjects in each group were pooled and a group survival curve was generated using standard statistical techniques [22]. Patients in the a.m. sleepier group showed a significant shift toward shorter bouts of sleep (*P *< 0.05) compared with the other groups. CFS, chronic fatigue syndrome.

Patients in the a.m. sleepier group showed significantly longer sleep latency, poorer sleep efficiency, and shorter duration of median sleep run than healthy controls (Table 3). The survival curve of all sleep runs depicted in Figure 1 shows that patients in the a.m. sleepier group had a lower percentage of long runs of sleep than the other two groups and healthy controls (that is, less continuous sleep). For example, the proportions of runs lasting more than 10 minutes were 39.3%, 45.5%, and 49.0% for patients in the a.m. sleepier group, the a.m. less sleepy group, and healthy controls, respectively. The difference in temporal distribution of periods of wakefulness is evident from the representative data in Figure 1. Both the control subject and the a.m. less sleepy patient have periods of wakefulness that are spaced more evenly over time than is the case for the a.m. sleepier patient, whose periods of wakefulness appear bunched in time (Figure 1a). The frequencies of these bouts occurring after sleep onset did not differ among groups (25 ± 15, 21 ± 5, and 23 ± 7 for patients in the a.m. sleepier group, in the a.m. less sleepy group, and for healthy controls, respectively).

The existence of coexisting FM did not predict sleep quality for patients (n = 7 in the a.m. less sleepy group and 5 in the a.m. sleepier group). Patients in the a.m. less sleepy group had significantly higher CES-D scores than those in the a.m. sleepier group (Table 3).

Prior to going to sleep, the a.m. less sleepy patient group reported more sleepiness than both healthy controls and patients in the a.m. sleepier group (*P *< 0.05). On the morning after their night in the sleep lab, patients in the a.m. sleepier group had significantly more sleepiness, fatigue, and pain than both healthy controls and patients in the a.m. less sleepy group (*P *< 0.05). Whereas fatigue and pain did decrease for patients in the a.m. less sleepy group, neither of these symptoms changed for patients in the a.m. sleepier group (Table 3). Visual analog scores for feeling blue showed minor differences among groups with little change after the night in the sleep lab (Table 3). Sleepiness and fatigue in both evening and morning were correlated (*P *< 0.05) in both patients and controls. Significant correlations were found for pain and fatigue reported in the evening and morning for patients but only in the evening for controls.

## Discussion

CFS is diagnosed using clinical criteria and is therefore probably comprised of a heterogeneous patient pool. The data reported here indicate that some CFS patients have a problem with normal regenerative sleep, which may be responsible for the genesis of their symptoms. In this study, we reduced patient pool heterogeneity by studying women only during a fixed period of their menstrual cycle and after excluding patients with either major depressive disorder or PSG-defined sleep disorders. As a group, the patients studied showed evidence for sleep disruption in the form of significantly reduced total sleep time, reduced sleep efficiency, and shorter bouts of sleep than healthy controls. In comparison with controls, sleep in CFS had little effect on either self-reported sleepiness or fatigue. And, interestingly, for patients only, ratings of sleepiness and fatigue correlated well with total sleep duration and efficiency.

Stratifying patients as to the presence of comorbid FM did not further reduce the heterogeneity seen in the patients' sleep structure. However, asking them about their level of sleepiness before they went to sleep and immediately after awakening did. Dichotomizing the patients into a group that felt sleepier after a night's sleep than before sleep and a group that felt less sleepy after a night's sleep reduced the variability of the sleep records considerably. Those patients reporting less sleepiness after a night's sleep had sleep structures similar to those for healthy controls except for a shorter total sleep time and a commensurate reduction in stage 2 sleep; moreover, they reported their fatigue and pain to diminish following sleep. In contrast, patients in the a.m. sleepier group had the greatest abnormalities of sleep architecture, including poor sleep efficiency, longer sleep latency, and more disrupted sleep as manifested by a higher percentage of short-duration sleep runs, than either controls or patients in the a.m. less sleepy group (Figure 1).

The net effect of this sleep disruption may be the genesis of symptoms reported by this group of CFS patients. The effects of sleep disruption are well known to produce severe daytime fatigue, an example being patients with sleep apnea who have very disturbed sleep. In the case of CFS, neither arousals nor periods of wakefulness *per se *may be the problem so much as the pattern in which they occur. Patients in the a.m. sleepier group had a shift away from longer bouts of sleep to more frequent short-sleep bouts (that is, fragmented sleep, which may prevent them from falling back to sleep after awakening), resulting in their developing fatigue, unrefreshing sleep, cognitive problems, and achiness. These data appear to support the sleep continuity theory, which hypothesizes that good sleep quality requires longer periods of uninterrupted sleep [24]. Reduced energy and cognitive problems are known to occur in healthy controls who have normal sleep time despite disrupted sleep produced experimentally [25]. In addition, some studies in healthy volunteers have reported increases in musculoskeletal pain and/or decreases in pain threshold after a period of sleep disruption or deprivation [26-28]. We are currently testing the hypothesis that the process responsible for disturbing the sleep of this group of CFS patients is an imbalance of the cytokine sleep network (that is, sleep-producing and sleep-disrupting cytokines) in favor of sleep-disrupting cytokines.

One purpose of this study was to determine whether stratifying our patient sample into those with and without comorbid FM would explain discrepancies in the literature as to rates of sleep pathology. It did not. Regardless of the presence of FM, our findings were similar to earlier reports of rather low rates of sleep pathology in CFS [6,7]. The low rates of sleep-disturbed breathing and PLMs we found in both patient groups are similar to those we found in our control group of sedentary women – rates that approached the values reported in the literature for unselected populations of healthy women [29,30]. However, in our hands, we found low rates of sleep-disturbed breathing for patients with CFS alone or CFS plus FM.

Importantly, the rates we found for sleep disturbed breathing include data along the entire spectrum of sleep disturbed breathing from overt sleep apnea to the upper airway resistance syndrome. The monitoring technique we used for airflow, a nasal cannula and examination of the flow signal for the characteristic shape of flow limitation, should have detected subtle forms of sleep disturbed breathing in our sleep studies, but only rare occurrences of patterns of air flow consistent with flow limitation were found here. Thus, although we did not use the more invasive technique of esophageal manometry to detect respiratory effort-related arousals, our results do not support an association between subtle forms of sleep disturbed breathing and CFS, even when co-morbid FM is present. This conclusion contrasts with an earlier report of EEG patterns "related to subtle, undiagnosed sleep-disordered breathing" in patients with chronic fatigue [31]. The apparent difference between these studies may relate to diagnostic specificity for CFS. All of our patients fulfilled the 1994 case definition for CFS, which requires their having disabling fatigue for at least 6 months plus at least four of eight infectious, neuropsychiatric, or rheumatological symptoms [15]; the subjects in the earlier study just had fatigue of long duration. Thus, our study does not eliminate the possibility that some patients with severe fatigue alone may have this problem as a result of subtle forms of sleep-disturbed breathing.

In summary, based on sleep patterns as assessed by polysomnography, patients with CFS alone and CFS plus FM have a similar rate of diagnosable sleep disorders; in fact, neither group has rates of sleep disorders higher than those found in healthy controls. Thus, sleep-disturbed breathing, narcolepsy, and leg movement disorders are an uncommon cause of medically unexplained fatigue or pain syndromes. Moreover, after excluding those patients from further analysis, CFS and FM patients have similar sleep structures. Our results also suggest that, even when the rate of arousals is within the normal range, sleep quality may be affected by a decrease in the length of episodes of uninterrupted sleep.

## Conclusion

CFS patients had significant differences in polysomnograpic findings from healthy controls and felt sleepier and more fatigued than controls after a night's sleep. This difference was due neither to diagnosable sleep disorders nor to coexisting FM but primarily to a decrease in the length of periods of uninterrupted sleep in the patients with more sleepiness in the morning than on the night before. This sleep disruption may explain the overwhelming fatigue, report of unrefreshing sleep, and pain of patients in this subgroup.

## Abbreviations

CES-D = Centers for Epidemiological Study-Depression; CFS = chronic fatigue syndrome; ECG = electrocardiogram; EEG = electroencephalogram; EMG = electromyogram; EOG = electrooculogram; FM = fibromyalgia; PLM = periodic leg movement; PSG = polysomnogram; RDI = respiratory disturbance index; REM = rapid eye movement; SWS = slow-wave sleep.

## Competing interests

The authors declare that they have no competing interests.

## Authors' contributions

FT provided interpretation of the results, statistical analyses, and preparation of the manuscript. BHN provided the design of the study, recruitment of the patients, organization and realization of the experimental design, interpretation of the results, and preparation of the manuscript. NSC provided the design of the study, interpretation of the results, and preparation of the manuscript. JF and CG provided recruitment of the patients, acquisition of data, and preparation of the manuscript. DMR assisted in study design, interpretation of the results, and preparation of the manuscript. All authors read and approved the final manuscript.
